# Predicting Algal Bloom Dynamics in Drinking Water Reservoirs Using High-Frequency In Situ Data and Machine Learning

**DOI:** 10.3390/toxins18050203

**Published:** 2026-04-28

**Authors:** Jiangbin Wang, Min Jiang, Shuhua Wang, Zixin Wang, Yikun Cui, Ying Feng, Shanshan Zhang, Mingjiang Cai, Yanping Zhong

**Affiliations:** 1 School of Resources and Environmental Sciences, Quanzhou Normal University, Quanzhou 362000, China; 2 Shanmei Reservoir Water Resources Allocation Center of Quanzhou, Quanzhou 362000, China; 3 Nantong R&D Center of Marine Science and Technology, Institute of Oceanology, Chinese Academy of Sciences, Nantong 226019, China

**Keywords:** phytoplankton abundance, drinking water reservoirs, optimized Transformer model, SHAP

## Abstract

Algal proliferation in subtropical drinking water reservoirs has become increasingly severe, and developing a reliable prediction for algal abundance through high-frequency in situ data is essential for early risk warning and effective management. This study analyzed the interannual variations in algal abundance in the Shanmei (SM) Reservoir, located in Quanzhou City, Fujian Province, China, based on the high-frequency data between 2020 and 2025, and forecasted algal abundance 24 h ahead via the optimized Transformer model. Results revealed that the SM reservoir exhibited seasonal variability in environmental factors, with persistently elevated pH during spring and summer, ranging from 7.12 to 9.66, and relatively high total nitrogen concentrations, ranging from 1.17 to 2.28 mg/L. Overall, algal abundance increased throughout the study period, and the annual average algal abundance in 2025 was 8.18 × 10^6^ cells/L, which was twice that in 2021. Model comparisons revealed that the optimized Transformer model exhibited the highest performance in terms of R^2^ = 0.88 when predicting the next hour using 12 days of data. Feature importance analysis based on SHapley Additive exPlanations (SHAPs) revealed that the predictions of algal dynamics were primarily influenced by previous-hours algal abundance, permanganate index, dissolved oxygen, air temperature, wind speed, and pH. This study revealed that the optimized independent learning model with integrated multi-scale features can significantly enhance the predictive performance of algal dynamics, offering a technical basis for early warning of algal blooms and refined reservoir management.

## 1. Introduction

An increasing number of lakes and reservoirs have been designated as centralized drinking water sources. In China, lakes and reservoirs accounted for 40.8% of centralized drinking water sources in 2023 [1]. Despite their critical role in water supply security, these systems are increasingly threatened by eutrophication and associated ecological degradation [2]. Long-term observations of 146 lakes larger than 10 km^2^ in Eastern China have revealed a pronounced deterioration trend, with the mean trophic index increasing by 8.2% from 1986 to 2020, a change strongly linked to intensified human activities such as urbanization and agricultural expansion [3]. Concurrently, satellite-based assessments have demonstrated pervasive warming of inland surface waters. Using a high-resolution global lake surface water temperature dataset, Tong et al. [4] have reported an average warming rate of 0.24 °C per decade between 1981 and 2020 across 92,245 lakes worldwide. These environmental changes have coincided with increasingly frequent and severe algal blooms, particularly cyanobacterial proliferations, driven by the combined effects of climate warming, nutrient enrichment, and watershed land-use alterations [5,6]. More than two-thirds of monitored lakes have experienced intensified summer algal bloom activity [7]. Certain bloom-forming cyanobacteria, such as *Anabaena flos-aquae* and *Aphanizomenon flos-aquae*, are capable of producing geosmin and 2-methylisoborneol, which are responsible for unpleasant taste and odor in drinking water [8,9]. Furthermore, some cyanobacteria (e.g., *Microcystis*) can produce potent hepatotoxins such as microcystins, which pose severe risks to human and animal health [10]. These metabolites not only impair esthetic water quality but also compromise aquatic ecosystem health and pose significant challenges to drinking water treatment processes. These issues underscore the urgent need to identify algal growth drivers and develop robust models for prediction, early warning, and targeted control in drinking water reservoirs.

The occurrence and development of algal blooms are driven by complex interactions among multiple environmental factors [2,10], including meteorological conditions, hydrological parameters, water temperature, and nutrient concentrations [7]. Consequently, algal abundance or biomass can be characterized as a multivariate, nonlinear function of these environmental variables, facilitating the approximation of complex patterns between environmental drivers and algal dynamics [5]. In this context, the application of machine learning techniques enhances our ability to capture these nonlinear correlations within aquatic ecosystems, particularly in the context of predicting phytoplankton dynamics. Existing algal prediction models can generally be classified into process-based models and data-driven models [5,11,12]. Process-based models simulate algal dynamics by explicitly representing physical, chemical, and ecological process mathematical equations. These models typically integrate hydrodynamic, water quality, and aquatic ecological components, with algal growth sub-models forming the core of ecological simulations [13]. Many studies have relied on the Monod-type formulations, which incorporate the algal responses to limiting factors such as temperature, light intensity, and nutrient concentrations [14]. For example, Rowe et al. [15] developed a modeling framework that combined satellite observations of harmful algal bloom distribution with hydrodynamic simulations and meteorological forcing to generate short-term bloom forecasts. However, in practical applications, it is often necessary to modify or simplify certain structural components of these models, which can result in suboptimal predictive performance. In contrast, data-driven models focus on extracting patterns and relationships directly from observational datasets using statistical and machine learning techniques. While this enables them to capture complex nonlinear dependencies between environmental variables and algal bloom dynamics, it is important to note that these dependencies represent correlational structures rather than mechanistic ecological relationships [2,16]. A wide range of machine learning algorithms, including convolutional neural networks (CNNs), recurrent neural networks, artificial neural networks, support vector machines, random forests, and long short-term memory (LSTM) networks, have demonstrated significant potential in algal bloom prediction. For example, Recknagel et al. [13] have evaluated three representative algorithms, including extreme gradient boosting, LSTM-Attention, and a hybrid evolutionary algorithm, to predict algal blooms in Lake Müggelsee, southeastern Berlin, over short time scales. Their findings indicated that the hybrid evolutionary algorithm and extreme gradient boosting exhibited superior generalization performance at daily time scale data, whereas LSTM performed slightly better for hourly predictions. Recent studies indicated that hybrid deep learning frameworks integrating process-based models and data-driven models can significantly improve time series forecasting performance. For instance, Xie et al. [11] have developed a hybrid algal bloom prediction model that combined mechanistic estimates of algal growth potential with data-driven time series regression, achieving reliable predictions of algal dynamics in eutrophic waters.

Nevertheless, the performance of such models remains highly dependent on data quality and sample size, and their generalization ability may be compromised when monitoring data are sparse, noisy, or incomplete. Traditional manual sampling and laboratory analyses can provide accurate data but are constrained by low temporal resolution and high costs, limiting their ability to capture short-term and rapid dynamic processes in aquatic environments. Recent advances in situ and online monitoring technologies for algal blooms have significantly increased the availability of long-term, high-frequency time series data. These datasets typically include various parameters, including water temperature, turbidity, pH, nutrient levels, chlorophyll *a* concentrations, and phytoplankton abundance. The enhanced temporal resolution of such data enables algal dynamics to be predicted at finer time scales, offering more detailed insights into diurnal bloom variability and providing timely and precise support for aquatic ecosystem management [2,16].

Based on long-term in situ monitoring data collected from a representative subtropical drinking water reservoir in Fujian, China, this study aimed to develop an optimized Transformer model for algal bloom prediction. The main objectives were as follows: (1) to evaluate the long-term variations in algal abundance in the reservoir and its driving environmental factors using Random Forest models; (2) to predict future algal cell density at hour time scales using meteorological variables and routine water quality monitoring parameters within a machine learning framework, and (3) to quantify the relative importance of features for the algal abundance prediction with SHAP (SHapley Additive exPlanations). The proposed algal density prediction framework is expected to support the development of high-precision early warning systems for algal blooms in drinking water reservoirs, providing both theoretical insights and practical tools to safeguard water quality and drinking water security.

## 2. Results

### 2.1. Variations in Environmental Factors in SM Reservoir

**Figure 1:**
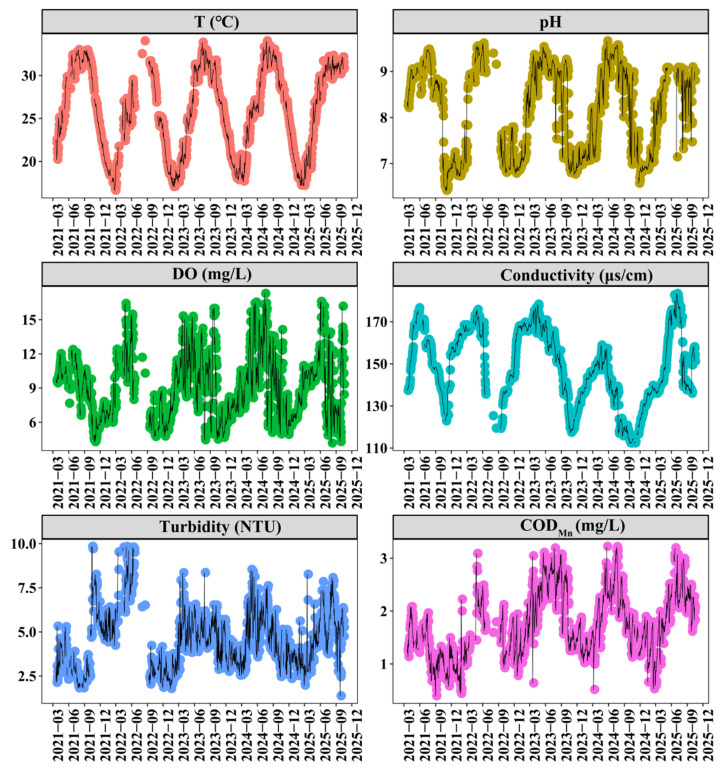
The variations in temperature, pH, dissolved oxygen (DO), conductivity, turbidity, and COD_Mn_ at the national monitoring points of Shanmei Reservoir from October 2020 to September 2025.

The SM Reservoir exhibited pronounced seasonal variability in multiple environmental parameters (Figure 1). Water temperature ranged from 16.60 to 34.00 °C (mean ± SD: 26.05 ± 5.06 °C), following a typical unimodal seasonal pattern, with maxima occurring in summer and minima in winter. pH values varied from 6.41 to 9.66 (mean ± SD: 8.24 ± 0.96), and closely tracked temperature dynamics, peaking in summer and declining during winter. The average DO concentration was 9.16 mg/L, while conductivity and COD_Mn_ averaged 147.87 μS/cm and 1.73 mg/L, respectively. These parameters exhibited comparable seasonal patterns, with marked increasing during late spring and early summer. Turbidity varied from 1.36 to 9.82 NTU (mean ± SD: 4.49 ± 1.73 NTU), generally showing higher values in summer and lower values in winter.

**Figure 2:**
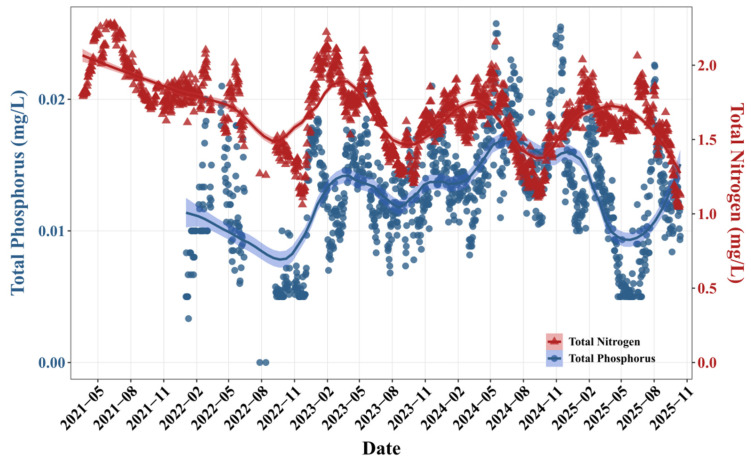
The variations in total phosphorus (TP) and total nitrogen (TN) at the national monitoring points of Shanmei Reservoir from October 2020 to September 2025. The lines are LOESS-smoothed trends, and the shaded areas indicate confidence intervals.

TP concentrations remained relatively low (mean ± SD: 0.010 ± 0.006 mg/L) in nutrient dynamics throughout the study period. In contrast, TN concentrations ranged from 1.05 to 2.28 mg/L (mean ± SD: 1.70 ± 0.26 mg/L) and showed a significant long-term decreasing trend (loess regression, *p* < 0.05) (Figure 2).

### 2.2. Dynamics in Algal Abundance and Its Influencing Factors

**Figure 3:**
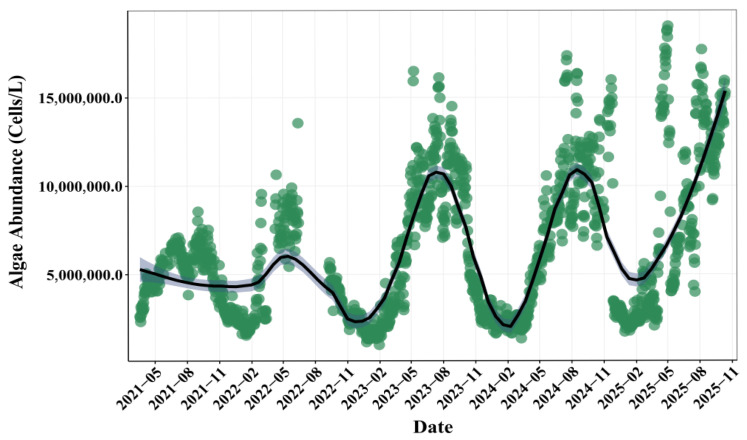
The variations in algal abundance at the national monitoring points of Shanmei Reservoir from October 2020 to September 2025. The lines are LOESS-smoothed trends, and the shaded areas indicate confidence intervals.

Analysis of monitoring data between 2020 and 2025 revealed that the averaged algal abundance in the SM Reservoir was 6.53 × 10^6^ cells/L (Figure 3). Over the study period, the algal abundance exhibited both complex intra-annual dynamics and seasonal variations. On an intra-annual scale, algal abundance remained relatively low and stable from 2021 to early 2023, followed by a marked increase beginning in the latter half of 2023. The algal abundance remained elevated with sustained fluctuations throughout 2024 to 2025. The annual average algal abundance in 2025 was 8.18 × 10^6^ cells/L, which was twice that in 2021. Seasonally, algal abundance in the SM Reservoir displayed a typical pattern, which was reflected by an increase beginning in spring, a sustained peak during summer and autumn, and a decline to winter minima.

**Figure 4:**
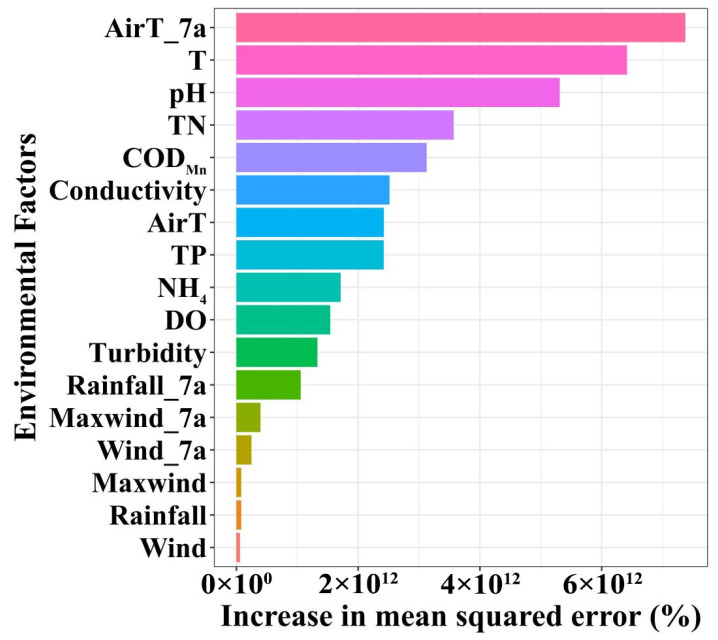
Order of increase in mean squared error based on Random Forest models.

To identify the key environmental factors driving these changes, a Random Forest models was used to evaluate feature importance (Figure 4). The results indicated that meteorological factors, particularly those related to thermal conditions, were the important predictors of algal abundance. Among these, the average temperature over the preceding seven days emerged as the most influential predictor, highlighting the role of short-term climatic fluctuations. Specifically, rising air temperatures directly or indirectly affected water temperature, stratification stability, and algal metabolic rates, thereby exerting a dominant influence on the algal dynamics in the reservoir. Among water quality variables, water temperature, pH, and TN concentration were the most important, confirming that the synergistic effects of water temperature and nitrogen nutrients remained fundamental mechanisms driving algal growth in the SM Reservoir. Additionally, the permanganate index also showed considerable importance, indicating that organic pollution load may further promote phytoplankton proliferation.

### 2.3. Performance Evaluation of the Transformer Model

**Figure 5:**
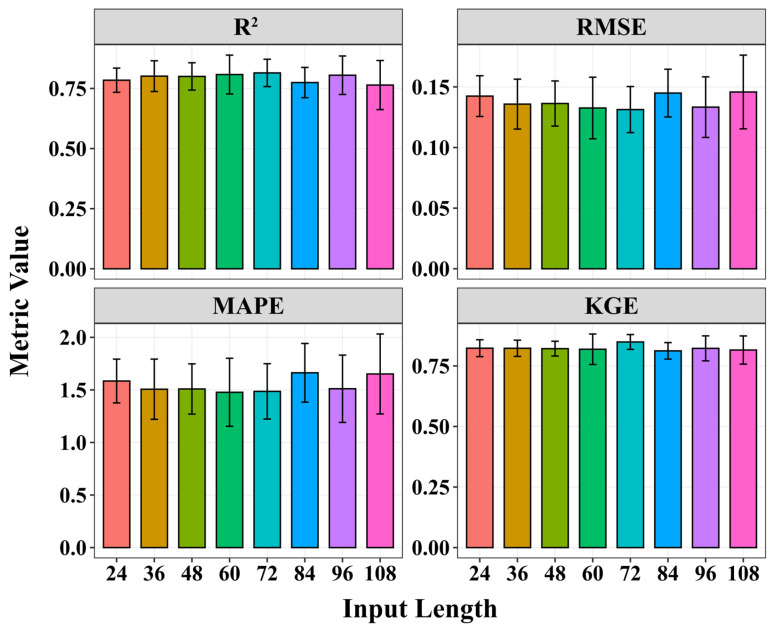
The predictive performance of Transformer model for different input sequence lengths.

The selection of input sequence length is crucial for the accuracy of time series forecasting. This study tested multiple input sequence lengths ranging from 24 to 108, with each length representing a 4 h interval. The model’s performance was evaluated based on R^2^, RMSE, MAPE, and KGE values, as illustrated in Figure 5. The model exhibited optimal overall performance with an input sequence length of 72, characterized by the lowest MAPE (1.486%), the lowest RMSE (0.131 cells/L), and the highest R^2^ (0.815). Furthermore, shorter data sequences offer practical advantages in terms of data availability and computational requirements. Therefore, this study established an input sequence length of 72 (equivalent to 288 h of historical data) as the optimal length for predicting phytoplankton abundance dynamics.

**Figure 6:**
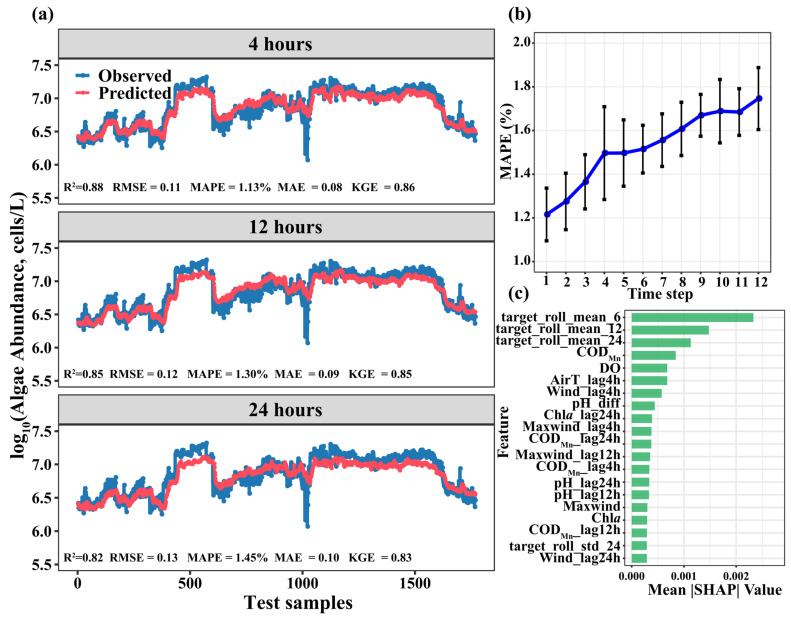
Display algal abundance prediction at 4, 12, and 24 h (**a**); detailed algal abundance predictions for using a 72 input sequence to forecast subsequent 12 sequences (**b**); SHAP analysis of key input features’ impact on algal abundance prediction based on the Transformer model (**c**).

To comprehensively assess the model’s predictive capability, the study evaluated forecast lengths of 1, 3, and 6 (i.e., 4, 12, and 24 h), with results presented in Figure 6. For the 4 h forecast, the model achieved the highest accuracy, with an R^2^ of 0.88, KGE of 0.86, RMSE of 0.11 cells/L, and MAPE of 1.13%. However, as the forecast horizon extended, the accuracy of the model’s predictions decreased. By the 24 h mark, R^2^ dropped to 0.82, KGE declined to 0.83, RMSE increased to 0.13 cells/L, and MAPE rose to 1.45%. Figure 6b showed the MAPE variation curve for the 12 forecast sequences (i.e., 48 h), indicating a gradual increase in prediction error. This trend further corroborates that the model does not replicate adjacent historical values as output predictions; otherwise, the forecast errors at each time point would remain relatively consistent.

To quantify the contribution of input variables to the prediction of algal abundance ahead of 24 h, this study calculated SHAP values based on the optimized Transformer model, with the mean absolute SHAP value (Mean |SHAP|) used as the metric for feature importance (Figure 6c). The results showed that the 6-step, 12-step, and 24-step rolling means of the target variable generally exhibited higher importances. Specifically, COD_Mn_ was the most influential feature, followed by DO concentration, air temperature, and wind from the previous day. Features from earlier time steps (e.g., 12 and 24 h) showed relatively lower importance.

## 3. Discussion

### 3.1. Key Factors Controlling Algal Abundance

The physicochemical parameters in the SM Reservoir exhibited distinct seasonal variability (Figure 1). Water temperature, pH, and permanganate index displayed similar seasonal variation patterns, with peaks in summer. In contrast, DO and electrical conductivity reached their highest levels in late spring and early summer. It was noteworthy that pH levels repeatedly exceeded the standard limit during spring and summer. Although TP concentrations remained consistently low throughout the study period, TN concentrations persistently exceeded 2 mg/L (Figure 2). These patterns were consistent with previous field observations [17], supporting the reliability of the data in this study. Over the past five years, TN concentrations in the reservoir have shown a gradual declining trend, while TP concentrations have remained relatively stable (Figure 2). Concurrently, algal abundances have increased significantly (Figure 3).

Variations in algal abundance were regulated by multiple environmental factors [5,10]. Analysis based on the Random Forest models indicated that water temperature, COD_Mn_, pH, nutrient concentrations, air temperature, and wind speeds were key drivers affecting phytoplankton abundance in the SM Reservoir (Figure 4). Key environmental variables affecting the predictions of Transformer model, SHAP analysis was employed (Figure 6c). The high importance of target_roll_mean_6, target_roll_mean_12, and target_roll_mean_24 in the SHAP analysis indicated that the recent historical state of algal abundance—both its average level and its variability—served as the strongest predictor of future changes. This reflectd the strong temporal autocorrelation of algal dynamics and suggestd that algal population dynamics often exhibited a certain degree of continuity. COD_Mn_ was the critical variable affecting short-term prediction of algal blooms. COD_Mn_ was a commonly used indicator for assessing the degree of organic pollution in water bodies. Its concentrations were influenced by multiple factors, including domestic sewage discharge, livestock and poultry farming pollution, agricultural non-point source inputs, rainfall runoff, and even extracellular organic matter released by algal metabolism [18]. In this study, the uncoupling trend of COD_Mn_ and nutrients concentrations has suggested that it may be primarily driven by inputs from livestock and poultry farming pollution. Notably, since 2020, large-scale fish stocking has been implemented in the reservoir as a biomanipulation measure to control algal density, which has also altered the sources of organic pollutants in the water to some extent. The positive relationship between algal abundance and COD_Mn_ was confirmed by several studies [18,19]. This relationship reflected that phytoplankton were one of the important sources of COD_Mn_ in water bodies, and elevated COD_Mn_ concentrations, in turn, promoted algal growth. For example, Lin et al. [18] have found that COD was positively correlated with the phytoplankton community structures in Dianchi Lake. Different phytoplankton groups exhibited distinct preferences for optimal pH ranges [17]. pH was not only a product of algal photosynthesis but also exerts feedback regulation on algal growth rhythms by altering the availability of inorganic carbon forms and the bioavailability of nutrients in water, thus playing a role in algal bloom prediction. Air temperature and wind speed also played an important role, and the driving effect of meteorological conditions on algal bloom dynamics was validated [16]. Air temperature and water temperature exerted immediate effects by regulating algal physiological and metabolic rates. Air temperature has primarily influenced phytoplankton growth by directly regulating water temperature, which was a core driver of phytoplankton growth. Water temperature has fundamentally modulated key physiological processes such as algal enzyme activity, photosynthetic and respiration rates, and life cycles [20]. Several studies have shown that phytoplankton are more likely to thrive in warmer waters, approximately 25 °C [21], which explains the peak of algal abundance during the late spring and early autumn (Figure 3). These findings have directly confirmed the high sensitivity of algal growth to short-term environmental changes, which have verified that algal dynamics were predominantly influenced by recent environmental conditions within a timescale of hours to days [22].

Nutrient concentrations are also important for long-term prediction. Notably, the TP concentration in the SM Reservoir remained consistently low throughout the year, whereas the TN concentration persistently exceeded 2 mg/L (Figure 2). This resulted in an extremely high average TN/TP atomic ratio of 362, indicating that the SM Reservoir was under persistent phosphorus limitation. However, monitoring trends over the past five years have shown that algal abundance has increased significantly under the background of declining TN concentrations and low TP levels. This seemingly paradoxical phenomenon may reflect an adaptive succession process within the phytoplankton community. Field observations have revealed a substantial increase in filamentous cyanobacteria, particularly *Pseudanabaena* sp., as the dominant species in the reservoir in recent years [23]. Although the current TP concentration in the SM Reservoir was relatively low, phosphorus remained a potential limiting nutrient. Future changes in phosphorus loading should still be monitored to prevent the water body from evolving toward eutrophication.

### 3.2. Predictive Performance of the Optimized Transformer Model on Algal Abundance

The optimized Transformer model demonstrated excellent performance in short-term algal bloom prediction. Through comparisons across different input and output sequence configurations, the model successfully captured the nonlinear and non-stationary relationships embedded in the environmental data. Selecting the optimal input sequence length was a key factor affecting the accuracy of forecasting. Although longer input sequences can capture more historical data, they may also introduce additional noise, thereby negatively impacting model performance [16]. In this study, multiple input sequence lengths ranging from 24 to 108 were tested, and found that the model achieves optimal overall performance when the input sequence length is 72 (i.e., 288 h of historical data). This length provided a good balance between capturing long-term trends and mitigating the negative effects of noise. Furthermore, as the input sequence length varies, the model performance exhibited nonlinear fluctuations, further highlighting the importance of selecting an appropriate input window for time series forecasting. Zhang et al. (2025) have also reported similar findings: when using a hybrid model to predict chlorophyll concentrations in the Jiangdong Reservoir, an input sequence length of 384 h (i.e., 16 days) provided the optimal balance [16].

Additionally, forecast accuracy is generally negatively associated with the forecast horizon: longer horizons entail higher uncertainty, leading to a gradual decline in model performance over time. For the 4 h forecast, the model achieved the highest accuracy, with an R^2^ of 0.88 (Figure 6). However, as the forecast horizon increased, model accuracy decreased. This declining trend is consistent with previous time series forecasting studies [16,24], indicating that longer forecast horizons typically lead to larger errors due to increased uncertainty in time series predictions. Figure 6b presents the MAPE variation curve for 12 forecast sequences, showing a gradual increase in prediction error. This trend further corroborates that the model does not simply replicate adjacent historical values as output predictions; otherwise, the forecast errors at each time point would remain relatively consistent.

Despite the performance decay over longer horizons, the Transformer model demonstrated robust predictive capability. Even when the forecast horizon extended to 24 h, the R^2^ still reached 0.82. Compared with previously developed algal bloom prediction models—such as traditional statistical models (e.g., conventional algorithms and decision tree), machine learning approaches (e.g., random forest and support vector machines), and even some recurrent neural networks—this performance is notably higher, as many previous models rarely achieved an R^2^ above 0.7 for 24 h ahead forecasts [13]. This robustness can be attributed to the self-attention mechanism inherent in the Transformer, which enabled the modeling of long-range temporal correlations between key events (e.g., historical algal blooms) and periodic patterns [25]. This multi-scale feature fusion allowed the model not only to accurately predict the timing of algal peaks and troughs but also to precisely replicate the magnitude of their fluctuations. Furthermore, the model demonstrated minimal overfitting, indicating robust generalization capability and the capacity to capture the complex dynamic characteristics of algal variability in reservoirs.

### 3.3. Implications for the Reservoir Water Management

Accurate prediction of algal bloom dynamics is essential for the precise management of eutrophic water, particularly for mitigating the risk of cyanotoxin contamination in drinking water sources [26]. Excessive nutrient inputs readily drive rapid algal proliferation and can trigger bloom events [6,21], which may be accompanied by the production of potent cyanotoxins. The optimized Transformer model developed in this study has demonstrated excellent performance in predicting algal abundance in the SM Reservoir, highlighting its strong potential as an early-warning tool for practical water quality management. The input features—such as COD_Mn_, DO, pH, water temperature, air temperature, and wind speed—can be continuously and automatically collected via existing in situ buoy systems and standard meteorological stations. Within an early-warning framework, real-time data streams can be formatted into time-lagged feature sequences and fed directly into the pre-trained model for rolling prediction. Such a framework can be further extended to provide early warnings for potential toxin events, as cyanotoxin concentration is typically correlated with the toxic cyanobacterial biomass or abundance. This process was highly automatable, and because the parameters monitored were routinely measured in reservoir and water quality networks globally, the model exhibited strong feasibility and adaptability for deployment across different aquatic systems [27].

According to the SHAP analysis, the rolling mean of the target variable (algal abundance), along with the 4 h and 12 h lagged features of key water quality parameters and meteorological factors, generally exhibited greater predictive utility. This can be attributed to the cumulative nature of algal abundance dynamics and the inherent lagged effects of abiotic factors on algal growth. This implied that, in systems such as the SM Reservoir, short-term algal bloom risk was more accurately captured by real-time or near-real-time monitoring data rather than by medium- to long-term averaged indices. To support effective short-term forecasting (e.g., 1–3 days), monitoring networks should therefore prioritize high-frequency (at least daily) and low-latency data transmission for key driving variables such as water temperature and nutrient concentrations. The current model in this study operated on an hourly scale based on 4 h of input data. Future research could incorporate higher-frequency monitoring data (e.g., hourly) to develop next-day or even intraday nowcasting models. As an example, with hourly water quality and meteorological inputs, the model achieves real-time forecasting capability, delivering probabilistic predictions of algal dynamics on an hourly basis up to 24 h ahead to inform dynamic operational decision-making. The Transformer model was particularly well-suited for processing such high-resolution data due to its ability to fuse multi-scale features. In practical management applications, the model outputs could be combined with predefined risk thresholds to establish a tiered early-warning system and linked to corresponding emergency response protocols.

Furthermore, the model’s high sensitivity to TN concentration—combined with the perennially elevated TN levels in SM Reservoir—reinforces the necessity of prioritizing nitrogen pollution control in long-term reservoir management.

## 4. Conclusions

Analysis of five-year high-frequency monitoring data from Shanmei Reservoir revealed pronounced seasonal variability in environmental parameters, with pH persistently exceeding 9 during spring and summer. Total nitrogen, despite a declining trend since 2020, generally remained above 2 mg/L, while total phosphorus remained consistently low. Nevertheless, phytoplankton abundance increased over the study period. Random forest analysis identified water temperature, pH, COD_Mn_, air temperature, and nutrient concentrations as the primary drivers of algal dynamics. The optimized Transformer model was developed for one-day-ahead algal abundance forecasting, and the model achieved the best predictive performance for 4 h ahead algal abundance forecasting (test R^2^ = 0.88). The SHAP analysis of the optimal model revealed that the rolling mean of the target variable exerted the strongest influence on predictions, with 4 h lagged feature showing higher importance than 24 h lagged maximum wind speed—underscoring the dominant role of recent conditions in driving short-term algal dynamics. These findings demonstrate that the optimized Transformer model, by integrating multi-scale feature extraction, is well suited for predicting algal abundance using high-temporal-resolution data, providing a methodological basis for short-term forecasting and early-warning systems to support adaptive reservoir management.

## 5. Materials and Methods

### 5.1. Study Area

**Figure 7:**
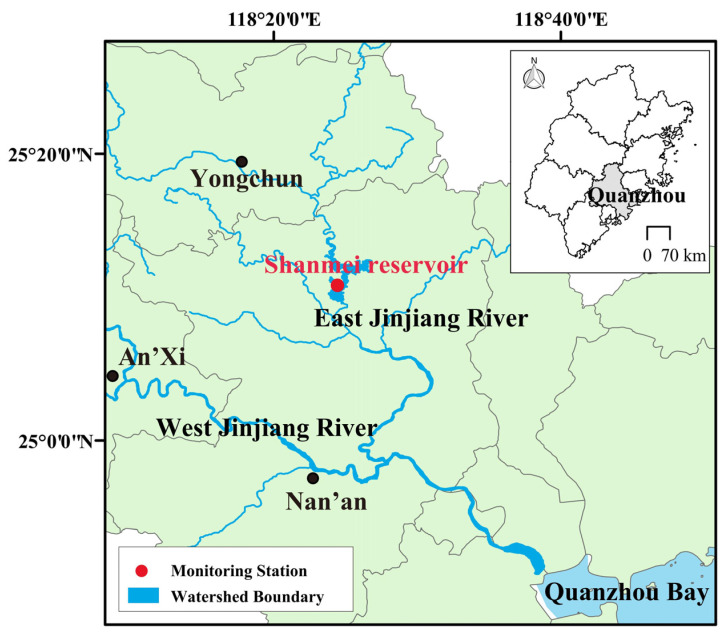
Location of the monitoring station in Shanmei Reservoir.

Shanmei (SM) Reservoir, located in Quanzhou City, southeastern Fujian Province, is in the middle reaches of the Dongxi River of the Jinjiang River basin (Figure 7). It serves comprehensive purposes, such as water supply, flood control, hydropower generation, and aquaculture. SM Reservoir was constructed beginning in 1985 and became operational in 1993. It has a total storage capacity of 656 million m^3^, of which 472 million m^3^ is effective storage capacity. The annual mean water depth varies from 15.3 to 46.6 m, classifying it as a typical deep-water drinking water source reservoir [26]. Its water quality security is directly related to the domestic, agricultural, and industrial water demands of more than 6 million people in the downstream Jinjiang region, as well as residents of the Kinmen area. In recent years, it has experienced increasing disturbance from human activities. Problems such as agricultural non-point source pollution, discharges from livestock and poultry farming, and unregulated rural domestic wastewater have become increasingly prominent, resulting in particularly severe exceedances of total nitrogen (TN) concentrations in the reservoir. Several studies have shown that the mean TN concentration in the reservoir exceeded 2.0 mg/L, while total phosphorus (TP) concentrations ranged from 0.013 to 0.032 mg/L [28,29]. Chlorophyll *a* concentrations in the vertical water varied substantially over time, ranging from 0.25 to 24.0 µg/L based on seven field campaigns conducted between 2022 and 2024. These values indicate a highly dynamic system with clear signs of eutrophication. The phytoplankton community was dominated by *Chlorophyta* and *Cyanophyta*, with *Pseudanabaena* sp. and *Microcystis* sp. identified as the dominant genera, whose dominance increased markedly during summer [23]. These characteristics collectively indicated that SM Reservoir has a substantial potential risk of algal bloom outbreaks [23]. Therefore, the development of a targeted algal bloom early-warning model is of great practical significance for safeguarding the water quality security of the reservoir.

### 5.2. Data Acquisition

The water quality data used in this study were obtained from the National Surface Water Quality Automatic Monitoring Real-time Data Publishing System. Continuous monitoring records were collected at the national control section of Shanmei Reservoir from 17 December 2020 to 31 October 2025, which were sourced from the National Surface Water Quality Automatic Monitoring Real-time Data Publishing System (https://szzqic.cnemc.cn:8070/GJZ/Business/Publish/Main.html). The monitoring station is located approximately 100 m upstream of the reservoir dam (Figure 7), with samples collected at a depth of 0.5 m. The monitoring strategy consisted of two distinct phases based on data frequency. From 17 December 2020 to 10 September 2021, measurements were recorded once per day. From 11 September 2021 to 31 October 2025, the monitoring frequency was increased to once every four hours. This high-frequency scheme was maintained consistently throughout this extended period, providing a robust dataset for capturing both seasonal patterns and short-term fluctuations in water quality parameters.

The monitored parameters included water temperature (WT), pH, dissolved oxygen (DO), conductivity, turbidity, chemical oxygen demand (COD_Mn_), TP, TN, Chlorophyll *a* (Chl *a*) and phytoplankton abundance. To ensure data quality and reliability, a rigorous quality control procedure was implemented. Initially, anomalous values resulting from instrument malfunctions or external disturbances were identified and removed using the 3σ criterion. Subsequently, total phosphorus (TP) values that were missing at the start of the study were replaced with half of the detection limit for values below the detection threshold. Finally, daily mean values were calculated for all parameters to standardize the temporal resolution of the dataset. Meteorological factors, including daily air temperature, rainfall, wind speed, maximum wind speed, and their average values over the preceding seven days, were sourced from the Meteorological Station Service Center of Shanmei Reservoir.

### 5.3. Model Construction

#### 5.3.1. Subsubsection

In constructing the model, data on environmental factors, meteorological variables, and algal abundance were collected at 4 h intervals from 11 September 2021 to 31 October 2025. The time resolution was set at 4 h. The raw dataset comprised 9186 samples with 13 original features: water temperature, pH, dissolved oxygen, conductivity, turbidity, permanganate index, total phosphorus, total nitrogen, chlorophyll *a*, air temperature, rainfall, wind speed, and maximum wind speed. To capture the temporal autocorrelation and dynamic trends of the target variable (algal abundance), rolling mean and standard deviation statistics with 6, 12, and 24 window sizes (equivalent to 1, 2, and 4 days) were calculated. Additionally, lagged features (4 h, 12 h, 24 h) of key water quality parameters (COD_Mn_, DO, pH, Chl *a*) and meteorological factors (air temperature, wind speed, maximum wind speed) were constructed. After incorporating lagged and interaction features, a total of 62 features were generated. For the feature variables, missing values were imputed using linear interpolation. Outliers were identified and addressed according to the 3σ principle. Min-max normalization was applied to eliminate dimensional discrepancies among variables, ensuring that both feature and target variables were standardized while maintaining their respective distribution characteristics.

During the training phase, the model was constructed using the first 80% of the data. To ensure the reliability of model performance and to avoid overfitting, 5-fold cross-validation was employed. The 80% of the data was divided into five subsets, with one subset selected as the validation set in each iteration, while the remaining four subsets were used as the training set, allowing for iterative validation. After each round of validation, the remaining 20% of the data was used as a test set for performance evaluation. The predictive performance of all models was assessed based on the average results from the 5-fold cross-validation on the test set.

#### 5.3.2. Model Selection

Several studies have employed CNN, LSTM and attention-based models to predict algal abundance and achieved promising predictive performance [11,13]. CNNs are specialized neural networks designed to extract local spatial and sequential features. Their primary advantages lie in local receptive fields and parameter sharing, which significantly reduce computational complexity while enhancing the generalization capability of feature extraction. The typical CNN architecture consists of convolutional layers, pooling layers, and fully connected layers [30]. LSTM networks are an advanced form of recurrent neural networks specifically designed to address the vanishing and exploding gradient problems encountered in conventional RNNs. LSTMs are well suited for modeling long-term temporal dependencies in sequential data [13]. The Transformer model, originally proposed by Vaswani et al. [31], is a deep neural network based on the self-attention mechanism. Unlike RNNs and CNNs, Transformers rely entirely on attention mechanisms to capture global dependencies within sequences, enabling parallel computation and significantly improving efficiency for long sequence modeling.

**Figure 8:**
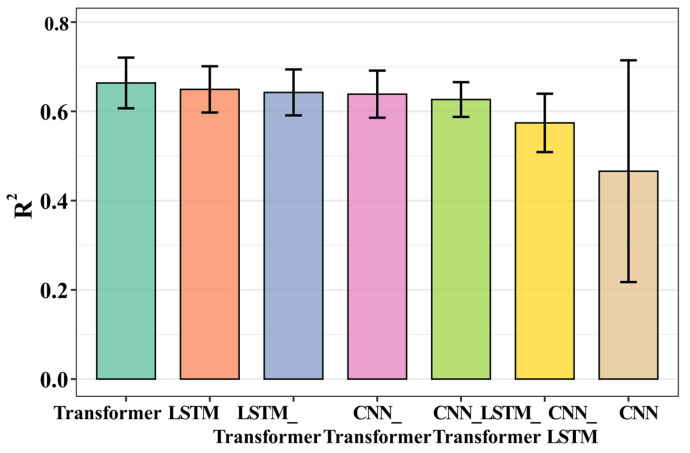
The performance of CNN, LSTM, Transformer, CNN_LSTM, CNN_Transformer, LSTM-Transformer and hybrid CNN_LSTM_Transformer models.

Based on the above network architectures, we constructed individual models (CNN-only, LSTM-only, Transformer-only) as well as their parallel hybrid models (e.g., CNN_LSTM, CNN_Transformer, LSTM_Transformer, and CNN_LSTM_Transformer). Before finalizing the model, we conducted an ablation study to evaluate each configuration under identical experimental settings, aiming to select the optimal model for algal abundance prediction. The specific hyperparameters were as follows: the input sequence length was 90, and the predict sequence length was 12. The model was trained for a maximum of 150 epochs, with early stopping implemented if the validation accuracy did not improve for 30 consecutive epochs. The learning rate was set to 0.00005, and the dropout rate was set to 0.4. The number of channels in the CNN model was set to 16, the hidden layers of the LSTM model was set to 32, and the dimension, number of heads, and number of layers in the Transformer models were 32, 2, and 1, respectively. The predictive performance of all models was evaluated using the coefficient of determination (R^2^) (Figure 8). The results showed that among the individual models, the Transformer performed best. Therefore, subsequent model optimization and data prediction were carried out based on the Transformer.

To further verify whether the proposed model truly captures the underlying patterns in the data rather than yielding deceptively high performance due to dataset simplicity, we supplemented the analysis with a random guessing baseline, as shown in the figure below. The negative R^2^ value of the random guessing predictions (R^2^ =−6.2657 ± 0.0661) indicated that its performance is even worse than simply using the mean of the training set as the forecast, failing completely to capture the variation in algal abundance.

#### 5.3.3. Hyperparameter Optimization

The input sequence length is another critical hyperparameter. In this study, a range of sequence lengths from 20 to 108 (equivalent to 4 to 18 days, with a time step of 4 h) was systematically evaluated. For each input sequence length, hyperparameters such as learning rate, dropout rate, and the number of attention heads were meticulously adjusted to ensure that the model achieves optimal performance for each sequence length.

At the optimal input sequence length, the remaining hyperparameters were fine-tuned using an empirically guided trial-and-error approach. The model was trained for a maximum of 200 epochs, with early stopping implemented if the validation accuracy did not improve for 20 consecutive epochs. The gradient was clipped to a maximum of 1.0, and the dropout rate was adjusted between 0.001 and 0.1, with 0.001 identified as the optimal value. The learning rate was set to 0.00005. To mitigate overfitting, a weight decay of 10^−3^ was applied; to enhance generalization, a dropout rate of 0.1 was utilized. The Transformer architecture consisted of a feature vector dimension of 32, two attention heads, and a single Transformer encoder layer.

To assess the accuracy of the model predictions, this study employed the R^2^, root mean square error (RMSE), mean absolute percentage error (MAPE), and Kling-Gupta efficiency coefficient (KGE) to evaluate the discrepancies between the predicted values and the observed values. SHapley Additive exPlanations (SHAP) values were computed for the optimal Transformer model. The best-performing Transformer model from cross-validation was loaded and set to evaluation mode. A random subset of 100 samples was selected from the test set as the background dataset, and another 200 samples were taken from the test set as the samples to be explained for SHAP value computation. Features were then sorted in descending order by their mean absolute SHAP values, and the top 20 most influential features were selected for further analysis.

## Data Availability

The original contributions presented in this study are included in the article. Further inquiries can be directed to the corresponding authors.

## References

[1] Zhang Y., Deng J., Zhou Y., Zhang Y., Qin B., Song C., Shi K., Zhu G., Hou X., Zhang Y. (2024). Drinking water safety improvement and future challenge of lakes and reservoirs. Sci. Bull..

[2] Liu Y., Yang B., Xie K., Sun J., Zhu S. (2025). Dongting Lake algal bloom forecasting: Robustness and accuracy analysis of deep learning models. J. Hazard. Mater..

[3] Hu M., Ma R., Xiong J., Wang M., Cao Z., Xue K. (2022). Eutrophication state in the Eastern China based on Landsat 35-year observations. Remote Sens. Environ..

[4] Tong Y., Feng L., Wang X., Pi X., Xu W., Woolway R.I. (2023). Global lakes are warming slower than surface air temperature due to accelerated evaporation. Nat. Water.

[5] Chen S., Huang J., Huang J., Wang P., Sun C., Zhang Z., Jiang S. (2025). Explainable deep learning identifies patterns and drivers of freshwater harmful algal blooms. Environ. Sci. Ecotechnol..

[6] Du Y., Zhao H., Li J., Mu Y., Yin Z., Wang M., Hong D., Zhang F., Wang S., Zhang B. (2024). Cyanobacterial blooms prediction in China’s large hypereutrophic lakes based on MODIS observations and Bayesian theory. J. Hazard. Mater..

[7] Naderian D., Noori R., Kim D., Jun C., Bateni S.M., Woolway R.I., Sharma S., Shi K., Qin B., Zhang Y. (2025). Environmental controls on the conversion of nutrients to chlorophyll in lakes. Water Res..

[8] Wu D., Chen M., Shen A., Shi Y. (2024). Spatiotemporal dynamics of 2-methylisoborneol produced by filamentous cyanobacteria and associated driving factors in Lake Taihu, China. Harmful Algae.

[9] Cao T., Fang J., Jia Z., Zhu Y., Su M., Zhang Q., Song Y., Yu J., Yang M. (2023). Early warning of MIB episode based on gene abundance and expression in drinking water reservoirs. Water Res..

[10] Zahir M., Su Y., Chen Y., Shahzad Muhammad I., Ayub G., Rahman Sami U., Ahmed T., Ijaz J. (2024). Anthropogenic and Climate-Driven Changes on Harmful Algal Blooms in Two Chinese Reservoirs. Ecohydrology.

[11] Xie Y., Chen S., Zhou F., Wang J., Liu Y., Gao Y., Yan X., Deng K., Chen C. (2025). Development of a hybrid algal population prediction (HAPP) model by algae growth potential estimation and time series regression and its application in one reservoir in China. Water Res..

[12] Wang Y., Xu C., Lin Q., Xiao W., Huang B., Lu W., Chen N., Chen J. (2024). Modeling of algal blooms: Advances, applications and prospects. Ocean Coast. Manag..

[13] Recknagel F., Shan K., Adrian R., Köhler J. (2025). Early warning of harmful cyanobacteria blooms based on high frequency in situ monitoring and intelligible machine learning modelling: The case study of Lake Müggelsee (Germany). Water Res..

[14] Jiang Y., Song Y., Liu J., Liu H., Zang X., Ji Z. (2025). Machine learning assisted precise prediction of algae bloom in large-scale water diversion engineering. Desalination.

[15] Rowe M.D., Anderson E.J., Wynne T.T., Stumpf R.P., Fanslow D.L., Kijanka K., Vanderploeg H.A., Strickler J.R., Davis T.W. (2016). Vertical distribution of buoyant Microcystis blooms in a Lagrangian particle tracking model for short-term forecasts in Lake Erie. J. Geophys. Res. Oceans.

[16] Zhang Y., Wang Y., Chen J., Lin L., Xiao W., Huang B. (2025). Enhancing short-term algal bloom forecasting through an anti-mimicking hybrid deep learning method. J. Environ. Manag..

[17] Liang L., Deng Y., Wang W., Zhou S., Zhang L. (2024). Influences of lower pH on phytoplankton growth in alkaline lakes after water transfer: Insights from a coupled hydrodynamic–algal ecological model and experimental analysis. Environ. Res..

[18] Lin Y., Zhong W., Zhang X., Zhou X., He L., Lv J., Zhao Z. (2023). Environmental DNA metabarcoding revealed the impacts of anthropogenic activities on phytoplankton diversity in Dianchi Lake and its three inflow rivers. Ecol. Evol..

[19] Yu H., Zhang J., Yin Z., Liu Z., Chen J., Xu J., Gao Q., Liu J. (2024). A method for quantifying the contribution of algal sources to CODMn in water bodies based on ecological chemometrics and its potential applications. J. Environ. Chem. Eng..

[20] Savadova-Ratkus K., Grendaitė D., Karosienė J., Stonevičius E., Kasperovičienė J., Koreivienė J. (2025). Modelling harmful algal blooms in a mono- and a polydominant eutrophic lake under temperature and nutrient changes. Water Res..

[21] Huisman J., Codd G.A., Paerl H.W., Ibelings B.W., Verspagen J.M.H., Visser P.M. (2018). Cyanobacterial blooms. Nat. Rev. Microbiol..

[22] Ma L., Xiao W., Laws E.A., Bai X., Chiang K.P., Liu X., Chen J., Huang B. (2020). Responses of phytoplankton communities to the effect of internal wave-powered upwelling. Limnol. Oceanogr..

[23] Wang S., Liang X., Zhang S., Cai M., Xie Z., Lin L., Chen Z., Rao Y., Zhong Y. (2025). Dynamics of Phytoplankton Communities and Their Characteristics of Realized Niches in a Drinking Reservoir. Ecol. Evol..

[24] Wang L., Shan K., Yi Y., Yang H., Zhang Y., Xie M., Zhou Q., Shang M. (2024). Employing hybrid deep learning for near-real-time forecasts of sensor-based algal parameters in a Microcystis bloom-dominated lake. Sci. Total Environ..

[25] Qian J., Pu N., Qian L., Xue X., Bi Y., Norra S. (2023). Identification of driving factors of algal growth in the South-to-North Water Diversion Project by Transformer-based deep learning. Water Biol. Secur..

[26] Scavia D., Wang Y., Obenour D. (2023). Advancing freshwater ecological forecasts: Harmful algal blooms in Lake Erie. Sci. Total Environ..

[27] Zhang Q., Wang R., Qi Y., Wen F. (2022). A watershed water quality prediction model based on attention mechanism and Bi-LSTM. Environ. Sci. Pollut. Res..

[28] Xu Z., Ge L., Zou W., Lv B., Yang J., Chai Z., Guo X., Zhu X., Kao S.-J. (2024). The underestimated role of manganese in modulating the nutrient structure in a eutrophic seasonally-stratified reservoir. Water Res..

[29] Chang J., Zhang S., Zhong Y., Ding S., Zhang W., Huang Q., Ji S., Chi Y. (2025). Long-term and seasonal evaluation on environmental microbiology and water quality of Shanmei reservoir in southeast China. Environ. Pollut..

[30] Pyo J., Park L.J., Pachepsky Y., Baek S.-S., Kim K., Cho K.H. (2020). Using convolutional neural network for predicting cyanobacteria concentrations in river water. Water Res..

[31] Vaswani A., Shazeer N., Parmar N., Uszkoreit J., Jones L., Gomez A.N., Kaiser L., Polosukhin I. (2017). Attention Is All You Need. Adv. Neural Inf. Process. Syst..

